# Supplementary figures and images for: Ketonuria Is Associated with Changes to the Abundance of Roseburia in the Gut Microbiota of Overweight and Obese Women at 16 Weeks Gestation: A Cross-Sectional Observational Study
Source: Nutrients. 2019 Aug 8;11(8):1836. doi: 10.3390/nu11081836 (PMC6723895; doi:10.3390/nu11081836)

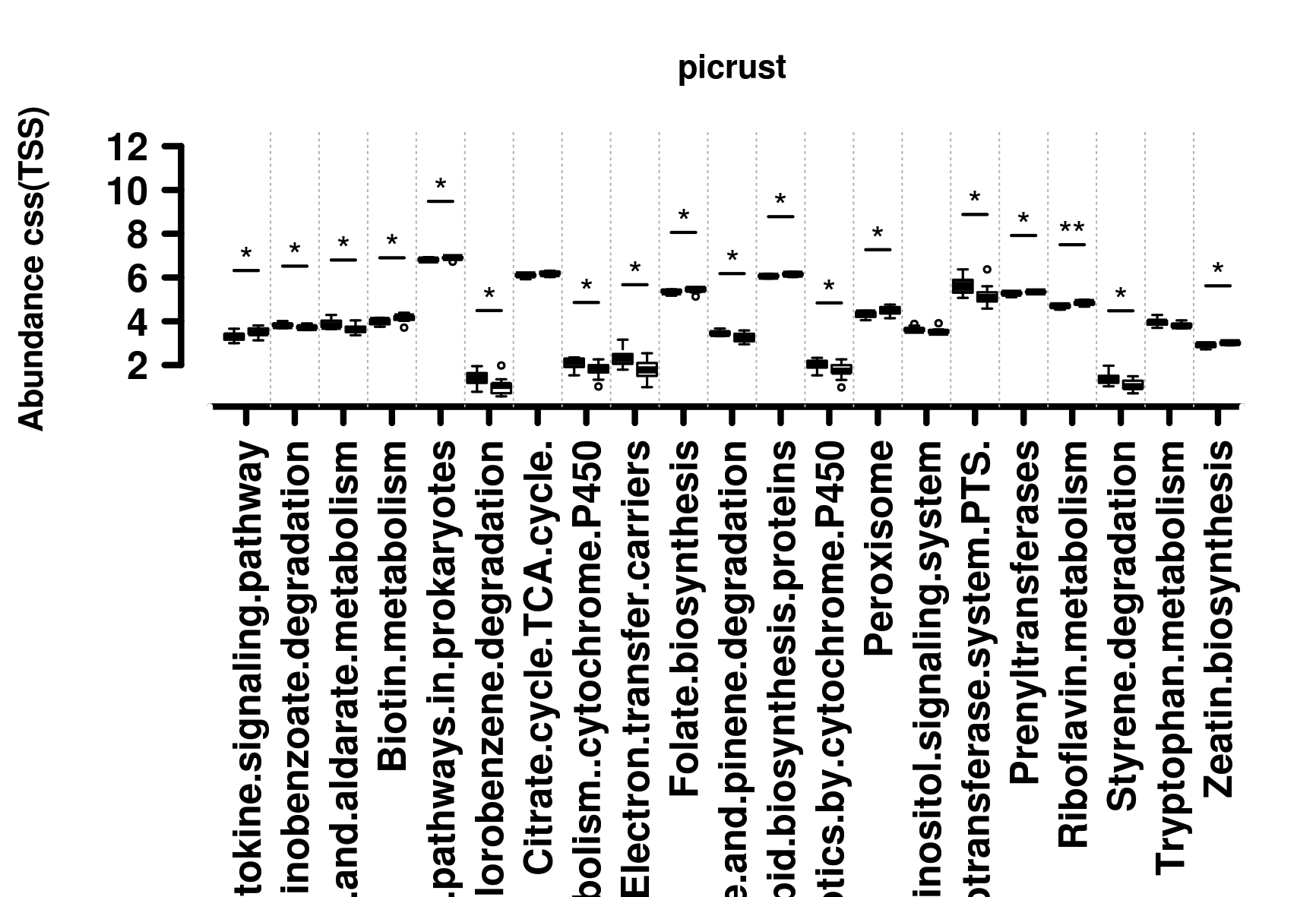


Figure S1 Predicted differential bacterial function in women with and without ketonuria

Supplement: Supplementary file 1 [file nutrients-11-01836-s001.zip › nutrients-564496-supplementary.docx]
